# Desafios da comunicação científica: o papel de *Cadernos de Saúde Pública* em 40 anos de história

**DOI:** 10.1590/0102-311XPT069924

**Published:** 2024-05-20

**Authors:** Nísia Trindade Lima

**Affiliations:** 1 Ministério da Saúde, Brasília, Brasil.; 2 Casa de Oswaldo Cruz, Fundação Oswaldo Cruz, Rio de Janeiro, Brasil.

Em 2014 tive o privilégio de escrever o Editorial comemorativo dos 30 anos de *Cadernos de Saúde Pública*, periódico de referência nacional e internacional para todos que se interessam pelo campo interdisciplinar da Saúde Coletiva. Passados 10 anos, sinto-me honrada e, sobretudo, desafiada ao ser convidada para compartilhar algumas ideias em um tempo que nos interpela a pensar o valor social da ciência e da saúde. E em *Cadernos*, conforme costumamos de forma íntima chamar essa potente revista, encontramos um acervo permanentemente atualizado para essa reflexão e tomada de posição. Hoje em todo o mundo, *pari passu* aos processos de mudança no mundo do trabalho, aos desafios ambientais, com as mudanças climáticas e seu impacto na saúde, aos processos de financeirização e à crise das democracias, convivemos com o negacionismo da ciência, seus valores, suas práticas e seus resultados. A COVID-19, um dos temas mais frequentes nas publicações em saúde nos últimos quatro anos, pode ser vista como importante marcador social da imbricação desses processos e os *Cadernos*, sem perda do rigor acadêmico e independência editorial, tornou-se importante e necessária tribuna em uma época em que se faz ainda mais necessário, e mesmo urgente, diferenciar autonomia científica de neutralidade quanto a valores ^1^. É o que se verifica particularmente nas seções Debate, Espaço Temático e Perspectivas.

Em uma visão retrospectiva lembro também do livro organizado por mim e colegas pesquisadores da Casa de Oswaldo Cruz, Fundação Oswaldo Cruz (COC/Fiocruz), para os 50 anos da Escola Nacional de Saúde Pública Sergio Arouca (ENSP/Fiocruz), em 2004 ^2^. Na Parte II (*Uma Casa de Pensamento e Ação*) da obra, no capítulo *Cadernos de Saúde Pública: Uma Trajetória de 20 Anos,* o leitor pode encontrar uma bem fundamentada descrição e interessante análise dos tempos de criação e consolidação da revista e aqui destaco a importância de Carlos Coimbra Jr., o seu mais longevo editor ^3^.

Interessante pensar em uma casa como metáfora para narrar essa história. Há uma vasta literatura nos campos da História das Ciências e dos Estudos Sociais da Ciência que aborda o papel dos periódicos científicos e uma de suas vertentes os analisa como instituições e não apenas como veículos de comunicação científica. Trata-se de vê-los como espaços de produção e reprodução da ciência, modelos de organização que contribuem para a formação de agendas científicas e sua comunicação para a sociedade ^4^. Por seu turno, a imagem da casa nos faz pensar no ambiente doméstico, espaço de afetos que se pode perceber na obra de criação institucional. Instituição acadêmica, casa e tribuna são três dimensões presentes em uma história, a um só tempo, intelectual e afetiva.

Desde 2012, *Cadernos de Saúde Pública* passou a incluir com maior ênfase a equidade de gênero e raça, a diversidade regional e maior representação internacional. Naquele ano, assumiram a editoria científica as pesquisadoras Marilia Sá Carvalho, Claudia Travassos e Cláudia Medina Coeli. Em 2015, Luciana Dias de Lima passou a compor o grupo de editoria científica e, após 2022, o trio de editoras é formado por ela, Luciana Correia Alves e Marilia Sá Carvalho, a última com 12 anos de dedicação a esse papel. A inclusão de uma pesquisadora de fora dos quadros da ENSP é outro fato digno de nota, além da valorização das mulheres em uma instituição na qual as barreiras de gênero ainda não foram superadas.

Os modelos clássicos de periódicos científicos datam do século XVII, e se firmaram como alternativa à comunicação científica anterior veiculada por cartas entre sábios ou em atas e memórias de sociedades científicas. Desde suas origens, a linguagem dos periódicos foi caracterizada pela impessoalidade e constituiu uma das características da própria construção do cientista como categoria social ^5^. A avaliação por pares, a padronização de referências, entre outras medidas que vemos hoje com um certo automatismo resultaram de complexos processos históricos. Hoje, muitos desses mecanismos se encontram em transformação com os *preprints*; processos mais coletivos de elaboração, indicando certamente a transição para novas formas de comunicação científica ^6^.

Também no que diz respeito à forma de comunicação científica, *Cadernos de Saúde Pública* foi pioneira em diferentes frentes, a exemplo do acesso aberto ao conhecimento científico e à contribuição para o portal SciELO. Nos últimos 15 anos, o periódico vem trazendo importantes debates sobre acesso aberto, além de aprofundar a discussão sobre avaliação acadêmica. Em seus artigos encontramos críticas argutas ao uso de indicadores como fator de impacto e argumentos consistentes sobre a necessidade de avaliações quantitativas. Destaca-se também a discussão sobre a pós-graduação no Brasil e seu papel na agenda científica e na resposta a questões prioritárias para a sociedade. Hoje a revista nos instiga a abordar temas como a ciência aberta, a transição digital e a inteligência artificial.

Em 2023, a revista abriu um Espaço Temático sobre a Amazônia, contribuindo entre, outras análises, para as relações entre saúde e sustentabilidade social e ambiental, além de trazer novas ideias sobre a região. Nesse ano, com a eleição do Presidente Lula e minha nomeação para o Ministério da Saúde retomou-se a interação do governo com a comunidade cientifica e a possibilidade de maior apropriação do conhecimento científico para a formulação de políticas públicas, além da retomada consistente do financiamento da pesquisa e do respeito à autonomia cientifica e ao pensamento crítico.

Um tema para reflexão: como pensar a Saúde Coletiva em tempos de transformação em um país no qual problemas de diferentes temporalidades parecem conviver: uma histórica desigualdade e os desafios do tempo presente; realidades sanitárias características do século XIX e problemas não superados no século XX, lado a lado com novos desafios do século XXI, a exemplo da pandemia de COVID-19? Pela sua tradição de ser simultaneamente espaço acadêmico, lugar de afetos e tribuna, espero que *Cadernos de Saúde Pública* continue a nos trazer a inquietação necessária e quem sabe novos caminhos para a comunicação da ciência e mesmo para o fazer científico.
